# Problematic Digital Technology Use Measures in Children Aged 0 to 6 Years: Scoping Review

**DOI:** 10.2196/59869

**Published:** 2025-03-18

**Authors:** Špela Selak, Janja Horvat, Mark Žmavc

**Affiliations:** 1 National Institute of Public Health Ljubjana Slovenia

**Keywords:** scoping review, measures, problematic digital technology use, children, early childhood, mobile phone

## Abstract

**Background:**

In the interest of accurately assessing the effects of digital technology use in early childhood, researchers and experts have emphasized the need to conceptualize and measure children’s digital technology use beyond screen time. Researchers have argued that many patterns of early digital technology use could be problematic, resulting in the emerging need to list and examine their measures.

**Objective:**

We aimed to review existing empirical literature that is using measures for problematic digital technology use in preschool children with the end goal of identifying a set of reliable and valid measures, predicting negative outcomes for children’s health, development, or well-being.

**Methods:**

We conducted a scoping review across the Web of Science, PubMed, and Google Scholar databases to identify peer-reviewed publications that were published from January 2012 to December 2023, were written in the English language, described an empirical study, and included a measure of problematic digital technology use beyond exposure (ie, screen time) in children aged 0 to 6 years.

**Results:**

The search yielded 95 empirical studies, in which 18 composite measures of problematic use and 23 measures of specific problematic use aspects were found. Existing composite measures conceptualize problematic use as either a group of risky behaviors or as a group of symptoms of a presumed underlying disorder, with the latter being more common. Looking at their conceptual background and psychometric properties, existing composite measures fall short of reliably assessing all the crucial aspects of problematic digital technology use in early childhood. Therefore, the benefits and shortcomings of single-aspect problematic digital technology use measures are evaluated and discussed.

**Conclusions:**

On the basis of current research, early exposure to digital technologies, device use before sleep, and solitary device use represent measures that have been consistently associated with negative outcomes for children. In addition, potential measures of problematic use include device use during meals, device use for emotional regulation, device multitasking, and technoference, warranting further research. Public health benefits of defining problematic digital technology use as a group of risky behaviors rather than a group of addiction symptoms are discussed.

## Introduction

### Background

Children are growing up in environments that have become increasingly saturated with various digital devices. According to a 2020 report on media use of children aged 0 to 8 years in the United States [1], 46% of those aged 2 to 4 years and 67% of those aged 5 to 8 years own a mobile device (ie, tablet or smartphone). The average daily screen time is 2.5 hours for children aged 2 to 4 years and 3.1 hours for those aged 5 to 8 years. The same data show that many children are being regularly exposed to screens even earlier; on average, they spend 49 minutes per day looking at a screen in the first 2 years of their lives. In addition, most of this screen use occurs in the absence of parents, with the likelihood of parental coviewing drastically decreasing with increasing age. The data also show that nearly three-quarters of screen time is spent watching video content, while reading, homework, and video chatting represent only 5% of children’s screen time. Furthermore, the data from the United Kingdom confirms that children in the United Kingdom are no exception since 48% of those aged 3 to 4 years and 57% of those aged 5 to 7 years owned a tablet in 2020, while 90% of those aged 3 to 4 years and 88% of those aged 5 to 7 years watched video-on-demand content [2].

Many stakeholders have expressed concerns about the potential harms associated with excessive screen use in early childhood. In 2016, the American Academy of Pediatrics issued a policy statement concerning media use in early childhood [3], recommending that children aged <18 to 24 months avoid digital media altogether. They argued that children aged <2 years require “...hand-on exploration and social interaction with trusted caregivers to develop their cognitive, language, motor, and socio-emotional skills,” while they cannot learn from traditional digital media in the same way. They stated that children aged 2 to 5 years should limit screen use to 1 hour per day of high-quality screen time, coviewed by parents to help them understand what they are seeing. The guidelines on screen use in children, published in other countries (eg, World Health Organization guidelines [4], Canadian guidelines [5], and Indian guidelines [6]), mostly followed the daily screen time limit recommendations for each age group.

However, the screen time restriction approach has been criticized, as many researchers and experts [7] argue that the existing scientific evidence is not conclusive enough to suggest appropriate amounts of screen use or preferable web-based activities for children of different ages. A brief look at systematic literature reviews on the effects of screen exposure on various outcomes in those aged 0 to 6 years reveals largely correlational evidence of the effect on adiposity, obesity, or BMI [8-10]; cognitive development [8,11]; psychosocial health [8]; and sleep duration [10]. However, to our knowledge, this evidence is yet to provide support for the suggested thresholds above which screen time is reliably detrimental to children. In a large study on 19,957 parents of children aged 2 to 5 years, authors found no empirical support for well-being benefits for children following the American Academy of Pediatrics screen time recommendations [12]. Researchers have argued that screen time, defined as the duration of exposure to digital devices, misses the content and context of digital technology (DT) use and is likely too broad and simplistic to be used as a stand-alone measure [13-15].

Owing to the shortcomings of screen time, the concept of problematic media use, defined as “...excessive use that interferes with the child’s functioning” [16], has gained momentum in the scientific community (eg, [17-19]). The focus on impairments in functioning is crucial to differentiate normal variation from a pathological level of behavior [16]. First, while we concur with the emphasis on functioning impairments, we will instead be referring to problematic DT use (PDTU), since the term “media” can also refer to traditional means of communication (eg, newspaper and radio) or collective institutions engaged in mass communication [20], both of which are erroneous interpretations of the concept. Second, we emphasize that although interference with functioning can certainly happen due to “excessive use” of DT, both the content and context of children’s DT use are arguably just as problematic. Thus, we suggest defining PDTU as any pattern of DT use that interferes with the child’s functioning.

Although the importance of content and context may seem rather obvious, contemporary research practices implied otherwise; a systematic review of 622 screen use measures in children aged 0 to 6 years [21] found that only 10.8% of these measures considered content and only 7% considered coviewing (ie, a measure of context). Importantly, this preference for exposure measures does not extend to older children and adolescents; a scoping review of empirical studies published between 2014 and 2019 by Browne et al [22] identified 162 measurement tools of DT use in children, adolescents, and young adults, most of these targeting problematic or excessive and addictive use beyond exposure. Among the 162 identified tools, only 5 were intended for preschool children, 3 of which came from gray literature. Evidently, despite many public concerns about harms associated with early DT use, most established and validated measuring tools of PDTU are intended for adolescents or adults, while instruments for young children are substantially less common. More recently, a systematic review by Rega et al [23] aimed to identify PDTU measures for children aged <10 years and found 9 parent report measurement tools aimed at children aged <6 years but did not analyze their content or psychometric properties. In conclusion, the various ways in which researchers have attempted to measure problematic media use in young children have not yet been critically reviewed and synthesized. In our view, this procedure is absolutely necessary to eventually arrive at comprehensive, valid, and cost-effective measures for children’s PDTU, which itself is a prerequisite for effective screening, prevention, and treatment of at-risk children.

### Objectives

On the basis of these insights, we aimed to review existing empirical studies, which included measures of PDTU in early childhood (ie, children aged 0 to 6 years) beyond screen time. Since our primary objective was to list and describe the various existing measures and operationalizations of the proposed concept (ie, PDTU) rather than answering a specific research question, we opted for a scoping review rather than a systematic review. The idea was to describe each measure or instrument in terms of its content, psychometric properties, and the negative outcomes it could lead to based on the results of each included study. Ideally, the goal was to arrive at a set of measures of PDTU for young children, which are psychometrically sound (ie, reliable and valid) and shown to be related to certain undesirable outcomes (eg, behavioral or emotional problems, deficiencies in terms of development, health, and well-being). Finally, we aimed to search beyond the developed measurement tools, seeking to identify the various single-item measures used by researchers to assess particular PDTU practices in preschool children.

## Methods

### Protocol and Registration

This study followed the PRISMA-ScR (Preferred Reporting Items for Systematic Reviews and Meta-Analyses Extension for Scoping Reviews) protocol [24]. The final version of the protocol was agreed upon by all authors and was not preregistered.

### Eligibility Criteria

The publications considered for review had to meet the following requirements: (1) peer-reviewed scientific publications; (2) published within the past 12 years (studies dating from January 1, 2012, to December 20, 2023); (3) written in the English language; (4) describing an empirical study; (5) including a measure of PDTU; and (6) conducted with the population of young children aged 0 to 6 years.

In addition, studies, which conceptualized and measured PDTU solely in terms of screen time, were excluded for reasons put forth in the Introduction section. Similarly, questions about which digital devices a child uses and to what extent (eg, minutes of use per day for each device type) were deemed overly simplistic, experiencing the same drawbacks as screen time. Finally, questions about the content the child usually engages in and to what extent (eg, minutes per day for each content type) were also not considered to be sufficient measures of PDTU. This is partly due to the vast variability of potential user experience that exists within a single content category (ie, “educational” content, video games, and cartoons). Furthermore, our concern was that due to the rapid evolution of content, any findings about the extent of the “problematic nature” of certain content types would likely soon be out of date (ie, as noted by Viner et al [25]).

### Information Sources

The search for relevant publications was conducted in the Web of Science, PubMed, and Google Scholar databases. Each of these databases was last searched on December 20, 2023.

### The Search Strategy

The final search strategy consisted of 4 groups of keywords, separated by the Boolean operator AND (Table 1). Each keyword group refers to a certain concept, which may be represented by any of the listed keywords, separated by the Boolean operator OR. In addition, filters for the date of publishing (ie, January 1, 2012, to December 20, 2023) and language (ie, the English language) were applied to all searches.

**Table 1:** The full search strategy used to obtain relevant records.

Concept	Keywords
Measure	scale OR questionnaire OR tool OR inventory OR measure OR instrument
Problematic	problem* OR excessive OR patholog* OR overus* OR addict* OR compulsive OR dependen*
Digital technology use	“screen us*” OR “screen exposure” OR “screen viewing” OR “screen watching” OR “screen behavio*” OR “media us*” OR “media exposure” OR “media viewing” OR “media watching” OR “media behavio*” OR “digital us*” OR “digital exposure” OR “digital play*” OR “digital behavio*” OR “device us*” OR “device exposure” OR “smartphone us*” OR “smartphone behavio*” OR “smart phone us*” OR “computer us*” OR “computer exposure” OR “computer viewing” OR “computer watching” OR “computer play*” OR “computer behavio*” OR “tablet us*” OR “tablet play*” OR “laptop us*” OR “TV us*” OR “TV exposure” OR “TV viewing” OR “TV watching” OR “TV behavio*” OR “television us*” OR “television exposure” OR “television viewing” OR “television watching” OR “internet us*” OR “internet exposure” OR “internet behavio*” OR “video game us*” OR “videogame play*” OR “game us*” OR “game exposure” OR “game play*” OR “game behavio*” OR “gaming”
Young children	child* OR infant* OR toddler* OR “pre-school*” OR preschool* OR kindergarten*

In each database, the final search strategy was applied only to titles and abstracts, as opposed to full texts, of published records. In the case of Google Scholar, a simplified search strategy was used due to the character limit, containing only the most common keywords for each of the 4 concepts. We developed the version of the search strategy with our backgrounds in psychology and psychometrics. Afterward, we scanned the records obtained, and the search strategy was adjusted accordingly. The adjustments mainly consisted of adding new keywords for each concept and adjusting the existing keywords (eg, shortening phrases to include alternative expressions). After multiple iterations, we derived the final search strategy, which yielded a manageable number of seemingly relevant publications in all databases.

### Selection of Sources of Evidence

All publication titles from the list of unique records were screened by 1 researcher using the inclusion and exclusion criteria. If eligibility was unclear based on the title, the abstract was read. When ambiguity remained (eg, age of children not specified), the publication was included for full-text review. In case of dilemmas, other authors were consulted, and disagreements were resolved collectively.

When screening titles, we included records mentioning the use of DTs of some kind (eg, internet use, gaming, and television viewing) among children. If the target population was described as “adolescents,” “teenagers,” “school-aged children,” or “students,” the record was excluded without reading the abstract. Studies of older children, adolescents, or young adults who assessed their DT use in early childhood retroactively were excluded. If the title mentioned the term “review” or “meta-analysis,” the study was excluded from our selection. If we discovered that a certain record does not refer to a peer-reviewed paper during the screening process, it was excluded from our selection. Titles referring to using DT for educational or therapeutic purposes were excluded from the selection. No automation tools were used for screening.

During the full-text review, studies on children outside the specified age range were excluded. Studies with mixed age samples were retained if measures for the target population were present, although outcomes and risk factor correlations were not reported, as they may not necessarily apply to children aged <6 years. Records were excluded if they used questionnaires that were not fully accessible; focused solely on screen time, devices, or content type; lacked key information; or were not in the English language.

### Data Charting Process

The first version of the data charting form, that is, its items and response categories for each item, was developed based on the study objective and was agreed upon by all authors. This version was pilot-tested by attempting to fill in the data for the first 20 full-text records on our list. On the basis of our findings, we made no changes to data items. However, we did adapt or add certain response categories for each item. The data charting was performed with the Excel (Microsoft Corp) software. Two researchers independently reviewed each eligible record and extracted data according to the previously established response categories in the charting form. Any inconsistencies regarding record eligibility or minor discrepancies in reported findings were identified and resolved on a case-by-case basis through discussions among the authors. While formal interrater reliability testing (eg, Cohen κ) was not conducted, we ensured consistency through regular meetings to discuss and resolve discrepancies, thereby maintaining a high level of reliability throughout the data extraction process.

### Data Items

The data from each eligible record, available in full text, were extracted according to the following items:

- Year of publishing
- Countries where data collection took place
- Developmental period: infants (ie, aged 0-1 year), toddlers (ie, aged 1-3 years), preschoolers (ie, aged 3-6 years), school-aged children (ie, aged 6-11 years), and adolescents (ie, aged >11 years)
- Population: the specifics of the population, other than age, for example, general population, children with attention-deficit/hyperactivity disorder, and children with dyslexia
- Sample size
- Study type: cross-sectional, longitudinal, quasi-experimental, and questionnaire development
- Format of instrument: survey, diary, observation, and interview
- Measures of PDTU: for example, use before sleep and early exposure
- Risk factors: demographic or population groups with more risk
- Adverse outcomes and correlates: outcomes associated with a certain aspect of children’s DT use. Only statistically significant findings are listed.
- Psychometric properties: reliability and any indicators of validity. Positive correlations with a separate PDTU measure (or screen time) serve as an indication of validity.

### Synthesis of Results

The evidence is presented in a table format, in 3 tables. Multimedia Appendix 1 [26-119] lists all papers included in the final selection and summarizes their key characteristics (eg, year of publishing, sample characteristics, and country) and the measures of PDTU that were used. Due to conceptual similarities among many single-item PDTU measures, we grouped them into categories (eg, age of first smartphone use, age of first television use, and use of DT before 1 year of age were classified as measures of early exposure).

## Results

### Selection of Sources of Evidence

**Figure 1:**
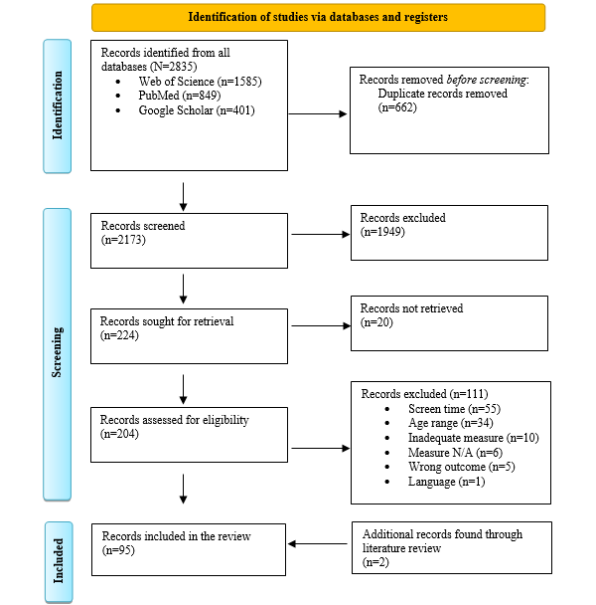
The PRISMA (Preferred Reporting Items for Systematic Reviews and Meta-Analyses) flowchart describing the process of selection of studies. N/A: not available.

The process of identifying papers that meet the eligibility criteria is shown in Figure 1.

### Study Characteristics

For each of the 95 identified papers, certain characteristics were extracted and summarized in a table format (Multimedia Appendix 1). In most (n=85, 89%) studies, PDTU measures were intended for preschool children. A total of 40 (42%) studies also considered toddlers as their target group, while only 7 (7%) studies included infants. A large majority (n=86, 91%) of studies were conducted with children with no particular specifics or deficits. Most (73/95, 77%) of the conducted studies could be described as cross-sectional, while only 6 (6%) were longitudinal. In addition, 6 (6%) studies used an experimental research design and a further 10 (10%) studies described questionnaire development. The findings of all longitudinal and experimental studies were discussed separately to assess the causal relationships of PDTU measures to relevant outcomes.

### Characteristics of PDTU Measures

#### Overview

In the 95 identified papers, a variety of aspects of PDTU were measured. Table 2 lists 23 distinct categories of PDTU measures found in the selected papers. It presents key characteristics for each measure for PDTU (eg, description and format), as well as all correlations with various risk factors and outcomes found in the papers.

**Table 2:** Existing measures for problematic digital technology use (PDTU) in children aged 0 to 6 years.

Measure	Description	Papers (N=95), n (%)	Format	Developmental period	Risk factors	Adverse outcomes and correlates	Correlation with other measures
Devices in the bedroom	The presence of digital devices in the room where the child sleeps	13 (14)	Survey	Infants, toddlers, and preschool children	Family income (–)a [26] Maternal education (–) [27]	Poor sleep quality (–), sleep problems, and emotional-behavioral difficulties [26] Obesity [28]	Screen time [27] Television viewing time [29]
Early exposure	Age at first exposure to digital screens or screen time before being aged 1 or 2 y	24 (25)	Survey	Infants, toddlers, and preschool children	Family income [26] Child’s age (–) [26,30,31] Maternal education (–) [32] Parental education [33] Autistic behavior [34] Autistic spectrum disorder [35] Traumatic experience [33] Family harmony (–) and parental anxiety or stress [31]	Poor sleep quality (–) [26] Sleep problems [26,33] Emotional-behavioral difficulties [26] Astigmatism risk [30] Cognitive developmentb (–) [32] Feeding problems and anger [33] Myopia [36] Social development (–) [37] Hyperactivity [31,38] Psychosomatic problems and psychological health (–) [31]	Problematic use [39] Screen time [32,40,41] Gaming [42]
Use during meals	Use of digital screens during mealtime	12 (13)	Survey	Infants, toddlers, and preschool children	—c	Weight status [29] Difficult temperament [43] Motor development (–) [44] Feeding difficulties and time spent eating [45]	Screen accessibility [46] Screen time [27]
Use before sleep	Frequency of digital device use before going to bed, typically within 1 h before bedtime	9 (9)	Survey and diary	Infants, toddlers, and preschool children	Family income (–) [47] Household chaos [48]	Self-regulation ability (–) [49] Late sleep timing, sleep problems, sleep duration (–), and sleep variability [47] Motor development (–) [50]	Restrictive mediation (–), instructive mediation, and couse [49]
Device multitasking	Exposure to ≥2 digital screen media simultaneously	2 (2)	Survey and interview	Infants, toddlers, and preschool children	Maternal education (–), paternal education (–), and positive parenting (–) [51]	Behavioral problems [51] Preschool cognitive ability (–) [51]	—
Restricted use	Parent’s use of rules and restrictions regarding the child’s use of digital media and whether the child obeys (also called restrictive mediation)	22 (23)	Survey and interview	Infants, toddlers, and preschool children	—	Behavioral problems [52] Executive functioning (–) [53]	Gaming [54] Problematic use (–) [55,56]
Background exposure	Amount of exposure to digital devices turned on in the background, while the child is occupied with other activities	12 (13)	Survey and observation	Infants, toddlers, and preschool children	Maternal education (–) [27]	Ability to delay gratification (–) and difficult temperament [43]	Parental television time [27] Screen time [27,57]
Problematic use	Various composite measures of problematic use of digital devices or technologies, including additional measures	27 (28)	Survey	Toddlers and preschool children	Male individual [39,58,59] Family income (–) [58,60,61] Unmarried [58,62] Non-White individuals [58] Parental age (–) [61,63] Parental education (–) [55,58,61,63] Nonworking mother [61,63] Single parent, extended family, family size, rural setting, day care use (–), and siblings [63] Age [56,61] Cognitive stimulation at home (–) [58] Parental efficacy (–) [56] Parental screen use and parental anxiety [64] Maternal stress [65]	Expressive vocabulary (–) [58,60] Phonological processing (–) [58,60] Emerging literacy (–) [58,60] Structural brain deficits in areas supporting language [60] Negative affect and effortful control (–) [62] Delaying essential needs [63] Behavioral problems and emotional intelligence (–) [66] Physical conflict [55] Parent-child relationship quality (–) [64]	Early exposure [39] Instrumental use [39] Emotional regulation [39,62] screen time [61,67,68] Television or video watching [68] Inconsistent mediation and restrictive mediation [55]
Screen time in one sitting	Amount of time on digital devices that the child spends in one sitting	2 (2)	Survey	Toddlers and preschool children	—	—	—
Delaying needs	Delaying daily needs during device use	2 (2)	Survey	Toddlers and preschool children	—	—	—
Educational use	Parents providing digital devices to their children to educate them	7 (7)	Survey	Toddlers and preschool children	—	Behavioral problems [52]	—
Use for entertainment	Parents providing digital devices to their children to entertain them	5 (5)	Survey	Toddlers and preschool children	—	—	—
Emotional regulation	Parents providing digital devices to their children to improve their mood or calm them down	12 (13)	Survey	Toddlers and preschool children	Parental education (–) [62,69] Male individuals and temperamental surgency [70]	Emotional difficulties andexecutive functioning (–) [70]	Problematic use [39,62] Emotional response [62] Screen time [41]
Instrumental use	Parents providing digital devices to their children to complete chores and tasks more easily and to distract them, using devices as a “babysitter”	11 (12)	Survey	Toddlers and preschool children	Age (–) [71]	Outdoor play (–) [72]	Problematic use [39]
Instructed use	Frequency of parents helping children understand the digital content through instructions and explanations during digital device use (also called instructive mediation)	7 (7)	Survey	Toddlers and preschool children	Age (–), male individuals (–), maternal education, and positive parenting [32]	Cognitive development [32]	Screen time and use before sleep [49]
Couse	The extent to which digital devices are used together with caregivers or siblings, as opposed to solitary use, sometimes as a ratio compared to total screen time	20 (21)	Survey and diary	Toddlers and preschool children	—	Language development delay (–) [73] Receptive language and expressive language [73,74] Emotional liability (–) [40] Social development [37]	Use before sleep [49] Screen time (–) [41,46,57]
Perceived negative effects	Parents’ perceived effect of child’s device use on their hobbies, concentration, social isolation, vision, hearing, appetite, sleeping, and family time	3 (3)	Survey and interview	Toddlers and preschool children	Autism [35]	—	—
Emotional reactivity	Frequency or intensity of child’s negative emotions as a response to devices being taken away or their use being limited	2 (2)	Survey and observation	Toddlers and preschool children	—	—	Emotional regulation and problematic use [62]
Self-regulation	Child’s lack of ability to disengage from activities on digital devices	1 (1)	Survey	Preschool children	—	—	—
Sensory regulation	Using screen media to help with sensory regulation by taking in or blocking out sensory input	1 (1)	Survey	Preschool children	—	—	—
Technoference	Frequency of interruptions or interferences in parent-child interactions due to child’s or parents’ use of digital devices	2 (2)	Survey	Preschool children	—	Behavioral problems [75,76] Psychosocial difficulties and frequency of parent-child interaction (–) [75] Prosociality (–) [76]	Screen time [75] Parents’ problematic use [75,76]
Conflict due to use	Conflict between parent and child because of or related to child’s device use	1 (1)	Survey	Preschool children	—	—	—
Concerns about use	Frequency of parent concerns regarding child’s digital technology use	2 (2)	Survey and interview	Preschool children	—	—	—

^a^The negative sign indicates a negative correlation between a risk factor or outcome and PDTU measure.

^b^Longitudinal associations are italicized.

^c^Data not available.

Of 95 studies, 29 (30%) used a composite measure of PDTU (ie, developed psychometric instruments and labeled problematic use), which contained multiple PDTU aspects within a single measurement tool. The most common among single-item measures used was early exposure (n=24, 25%), followed by restricted use (n=22, 23%) and couse (n=20, 21%).

In terms of measurement format, survey-based measures of PDTU were by far the most common. Each of the listed PDTU measures existed in survey format in at least 1 study. In total, 34% (8/23) of the PDTU measures used a nonsurvey format in at least 1 study. The data for 17% (4/23) of the measures were obtained through an interview, 9% (2/23) through a diary format, and 9% (2/23) through observation. For each PDTU measure, data were obtained on preschool children (aged 3 to 6 years) in at least 1 study. A large proportion (16/23, 78%) of measures was also applied to toddlers (aged 1 to 3 years). Less than a third (7/23, 30%) of all measures were used in studies, which included infants (aged 0 to 1 year).

#### Risk Factors

The most commonly reported demographic risk factor for PDTU was lower parental education (ie, either maternal or paternal). Of the 23 PDTU measures, negative associations with parental education were found for 7 (30%) measures or 70% (n=16) of all measures for which any risk factors were reported. In addition, being a male participant was found to be a risk factor for 3 (13%) measures and having autism spectrum was found to be a risk factor for 2 (9%) measures. The child’s age was a risk factor for 3 (13%) PDTU measures, although both lower and higher age were found to be risk factors, depending on the particular aspect (Table 2).

#### Detrimental Outcomes

PDTU measures were associated with 28 unique, undesirable outcomes. Of 23 measures, 11 (48%) were found to be associated with behavioral-emotional difficulties of children, while 7 (30%) were correlated with developmental outcomes (eg, deficiencies in language, cognition, or motor development). A total of 6 (26%) measures were associated with undesirable habits (eg, poor sleeping and eating habits or delaying needs), and another 3 (13%) measures were associated with physical outcomes (eg, obesity and vision problems).

#### Findings From Longitudinal Studies

A longitudinal study by Supanitayanon et al [32] showed that delayed introduction to DTs (a measure of early exposure) and verbal interaction during media use in the first 2 years of life (ie, a measure of instructive mediation) significantly predicted a child’s cognitive development at 2, 3, and 4 years of age. A longitudinal study by Srisinghasongkram et al [51] showed that screen media multitasking at 18 months (ie, device multitasking) is associated with decreased preschool cognition at 4 years and behavioral problems at 4 and 6 years. In a longitudinal study by Radesky et al [70], bidirectional cross-lagged correlations between using devices for calming purposes and emotional reactivity (ie, instability) were found, specifically in boys and children with higher temperamental surgency. A study by Coyne et al [56] showed a longitudinal association between 2 PDTU measures: restricted use was associated with lower problematic use. A study by Gueron-Sela et al [77] showed no longitudinal relationship among screen time, background exposure to DTs or using DTs for emotional regulation during lockdown periods, and children’s behavioral problems after lockdown.

### Instrument Characteristics

#### Overview

Among the measures of PDTU, we identified 18 multi-item measures of problematic use, which were either fully available or had their items described in sufficient detail. The most commonly used was Problematic Media Use Measure–Short Form (PMUM-SF), used in 9% (9/95) of the studies. All other instruments were used in 1 or 2 studies at most. All the identified instruments were applied to preschool children (aged 3-6 years) in at least 1 study. Only 22% (4/18) of them were also tested on toddlers (aged 1-3 years), while no instrument was used in studies on infants (aged 0-1 year). Table 3 summarizes important characteristics (eg, theoretical background and psychometric properties) of all measurement tools targeting PDTU.

**Table 3:** Characteristics of existing composite measures for problematic digital technology use (N=18).

Questionnaire	Description	Factors or content categories	Items, n	Background	Year	References	Reliability	Validity	Developmental period
Technology addiction scale	A parent report measure of technology addiction for children aged 2 to 5 y	Impulsiveness (impulsiveness symptoms of using a technological device) Implicit attitudes (emotional symptoms and reactions toward the use of a technological device)	9	Item pool was generated based on internet addiction symptoms (in adolescence)	2023	[78]	Cronbach α=0.90	CFA^a^: CMIN^b^ (*df*)=2.141, GFI^c^=0.964, CFI^d^=0.981, and RMSEA^e^=0.061	Toddlers and preschool children
DSEQ^f^ for young children	A parent report measure of screen exposure for children aged 2 to 5 y	Sociodemographic screen time exposure and home media environment Level of physical activity Media-related behaviors and parental perceptions domain	86	Questionnaire was developed based on existing tools, parent interviews, and expert interviews	2021	[79]	Cronbach α for each subdimension were 0.82 and 0.74. κ value=0.52-1.0 and ICC^g^=0.62-0.99	Good face and content validity as judged by 9 independent experts	Toddlers and preschool children
Seven-in-Seven Screen Exposure Questionnaire	A parent report measure of problematic screen exposure	Screen time Early exposure Use during meals Use before sleep Content Coviewing Restrictive mediation	7	Items were designed using the AAP^h^ recommendations for children’s media use	2021	[63]	Cronbach α=0.49	Use of touchscreens (+)^i^ and EFA^j^ found 3 factors	Toddlers and preschool children
Electronic Media Use Questionnaire	A parent report measure of how “serious” the electronic media use of the child is	Electronic media time management Interpersonal and health conditions caused by electronic media use Life conflicts arising from electronic media use Emotional experiences related to electronic media use	14	Questionnaire adapted from the Video Game Use Questionnaire by changing the term “video game” to “electronic media” and reducing item numbers based on factor analysis^k^	2023	[80]	Cronbach α=0.93, Cronbach α of each subdimension were 0.73, 0.80, 0.77, and 0.82	CFA: *χ*^*2*^=2.71, RMSEA=0.08, GFI=0.91, NFI^l^=0.91, IFI^m^=0.94, TFI^n^=0.92, and CFI=0.94	Toddlers and preschool and school-aged children
PMUM^o^	A parent report measure of child’s addictive use of screen media	Preoccupation Withdrawal Tolerance Unsuccessful attempts by parents to control use Loss of interest in previous hobbies and entertainment Deceived others about use Use to escape or relieve a negative mood Jeopardized/lost a relationship or had compromised functioning in school due to use Continued use despite psychosocial problems	27	Items were generated based on criteria suggested for IGD^p^ in the *DSM-5*^q^. Content used to generate items that correspond to the *DSM-5* criteria were drawn from the literature on problematic media use in adolescents, clinical experience, and interviews with mothers of children aged 4 to 8 y.	2019	[65,81]	Cronbach α=0.97	PMUM-SF^r^ (+), screen time (+), concerns about use (+), and instrumental use (+); PMUM predicts psychosocial functioning over and above screen time (24% of additional variance explained)	Toddlers and preschool and school-aged children
PMUM-SF	A parent report measure of child’s addictive use of screen media; a short version of PMUM	Preoccupation Withdrawal Tolerance Unsuccessful attempts by parents to control use Loss of interest in previous hobbies and entertainment Deceived others about use Use to escape or relieve a negative mood Jeopardized/lost a relationship or had compromised functioning in school due to use Continued use despite psychosocial problems	9	Items were based on the *DSM-5* criteria for internet gaming disorder	2019	[56,59,62, 64,67,73, 81-83]	Cronbach α=0.93, 0.80, 0.94, 0.90, and 0.91	PMUM (+), screen time (+), concerns about use (+), parent-child conflict over screen media use (+), emotional regulation (+), parental media efficacy (+), and parental screen addiction (+); PMUM-SF predicts psychosocial functioning over and above screen time; acceptable fit in CFA (RMSEA=0.085; CFI=0.961; SRMR^s^=0.024); and measurement invariance between boys and girls indicated that factor structure is the same for both groups.^t^	Toddlers, preschool and school-aged children, and adolescents
PMPUS^u^	A parent report measure of problematic mobile phone use	Deprivation Adverse outcomes Control problems Interaction avoidance	26	The original version of the scale was aimed at university students. Items were generated based on interviews/open-ended questions with students. PMPUS was adapted for children through a focus group discussion with experts.	2016	[39]	Cronbach α=0.90 (factors from 0.90 to 0.91)	Early exposure (–), Instrumental use (+), Emotion regulation (+); EFA found the listed 4 factors	Preschool children
PCIAT^w^	A parent report measure of child’s internet addiction	—x	20 (21)	The original version of the test was aimed at adolescents. A total of 5 items were removed as they were not relevant for preschool children.	2016	[52]	ICC=0.92	Panel of experts confirmed content validity (CVI=0.989)	Preschool children
SMALLQ^y^	A parent report measure of digital media habits of children, which can be used to monitor changes over time	Digital media environment at home Parent digital media habits Child digital media habits (ie, outside of preschool) Parent perception of digital media use Parent concerns Parent awareness of guidelines “Pon-digital (physical and playing) habits”	25	Items were based on reviewed literature, focus group, and cognitive interviews	2019	[84]	—	Acceptable face and content validity as judged by 4 independent experts	Preschool children
ScreenQ survey	A parent report measure of adherence to AAP recommendations for media use in childhood	Access to screens Frequency of use Content viewed Coviewing	15	The conceptual model for the ScreenQ survey was derived from aspects of media use cited in current AAP recommendations.	2020	[58,60]	Cronbach α=0.74	Criterion-related validity referenced to external standards of child cognitive skills and home cognitive environment	Preschool children
S-scale^z^ for children	A parent report measure used for screening of problematic smartphone use in children	Self-control failure Salience Serious consequences	9	The scale was based on the SAPS^aa^, which was developed based on clinical experiences, research findings, and previous diagnostic instruments.	2016	[61]	Cronbach α=0.80	Screen time (+), tv/video watching (+)	Preschool children
Addiction measurement tools of measuring smartphone addiction of children and adolescents	A parent report measure of child’s smartphone addiction tendencies	Interference with daily life Voluntary isolation Need for compulsory control Personality distortion	18	—	2011	[66]	Cronbach α=0.66 to 0.90 for factors	CFA loading levels between 0.59 and 0.80	Preschool children
PTUS-YC^ab^	A parent report measure of child’s problematic technology use	Continuity of use Resistance to control Effect on development Deprivation escape	26	Items were based on reviewed literature and criteria for internet addiction	2022	[85]	Cronbach α=0.94 (factors from 0.88 to 0.94)	EFA: 4 factors with factor loadings between 0.37 and 0.83. AVE for factors from 0.41 to 0.60. CFA: 4 factor structure – RMSEA=0.076, NFI=0.871, CFI=0.906, IFI=0.907, SRMR=0.071	Preschool children
SAS-SV^ac^	A parent report measure of proneness to problematic smartphone use	Daily life disturbance Positive anticipation Withdrawal Cyberspace-oriented relationship Overuse Tolerance	10	SAS (used for adolescents) was adapted to be suitable for young children	2013	[55]	Cronbach α=0.91	Inconsistent mediation (+), restricted use (–)	Preschool children
QQ-MediaSEED^ad^	A parent report on quantity and quality of digital media use for bilingual children aged 3 to 6 y	Demographics Digital media use Parental mediation	—	Inspired by existing tools	2022	[86]	Cronbach α for each subdimension were 0.83, 0.84, 0.90 and 0.84	Appropriate face validity as judged by stakeholders. PCA^ae^ shows two components “restriction” and “instruction, supervision, and co-use”	Preschool children
YC-CGD^af^	A parent report measure of computer gaming disorder symptoms in young children	—	11	Substance-related addiction criteria of *ICD-10*^ag^	2017	[82]	Cronbach α=0.83	Principal component analysis: One component solution, 38.2% variance explained. Acceptable PCA factor loadings	Preschool children, school-aged children
Video Game Engagement in Children Questionnaire	A parent report measure of video game engagement	Interest in the activity Focus during play Challenges in discontinuation Social disengagement	19	Original set of items reduced based on EFA and CFA results	2023	[87,88]	Cronbach α=0.93, Cronbach α of each subdimension were 0.86, 0.82, 0.80, and 0.86	EFA: 4 factors, CFA: CMIN/(*df*)=4.8, RMSEA=0.07, CFI=0.91, TLI^ah^=0.90, and SRMR=0.058	Preschool and school-aged children
SASC-P^ai^	A parent report measure of a child’s smartphone addiction	Smartphone dependence Psychological ill health Physical ill health Academic performance Social relationship Family relationship	24	Domains of smartphone addiction were proposed based on previous studies detailing the diagnostic criteria for smartphone addiction	2021	[83]	Cronbach α=0.97 (0.82-0.91 for domains), test-retest reliability was 0.96 (0.68-0.85 for domains)	PCA was used to establish domains. Each domain included items with>0.30 factor loadings. Content validity was examined by an expert panel of 4 psychiatrists and 4 psychologists. A focus group was identified to establish face validity.	Preschool and school-aged children and adolescents

^a^CFA: confirmatory factor analysis.

^b^CMIN: minimum discrepancy of confirmatory factor analysis.

^c^GFI: goodness of fit index.

^d^CFI: comparative fit index.

^e^RMSEA: root mean square error of approximation.

^f^DSEQ: Digital Screen Exposure Questionnaire.

^g^ICC: intraclass coefficient.

^h^AAP: American Academy of Pediatrics.

^i^(+): positive correlation.

^j^EFA: exploratory factor analysis.

^k^Questionnaire not accessible.

^l^NFI: normal fit index.

^m^IFI: incremental fit index.

^n^TFI: total fit index.

^o^PMUM: Problematic Media Use Measure.

^p^IGD: internet gaming disorder.

^q^DSM-5: Diagnostic and Statistical Manual of Mental Disorders (Fifth Edition).

^r^PMUM-SF: Problematic Media Use Measure–Short Form.

^s^SRMR: standardized root mean square residual.

^t^Data regarding the incremental validity, model fit, and measurement invariance of the PMUM-SF and PMUM were gathered on children aged 4 to 11 years and 4 to 14 years.

^u^PMPUS: Problematic Mobile Phone Use Scale.

^v^The negative sign indicates negative correlation.

^w^PCIAT: parent-child internet addiction test.

^x^Data not available.

^y^SMALLQ: Surveillance of digital media habits in early childhood questionnaire.

^z^S-Scale: Smartphone Overdependence Scale.

^aa^SAPS: Smartphone Addiction Proneness Scale.

^ab^PTUS-YC: Problematic Technology Use Scale for Young Children.

^ac^SAS-SV: Smartphone Addiction Scale-short version.

^ad^QQ-MediaSEED: Quantity and Quality of Media Screens in Early Education and Development.

^ae^PCA: principal component analysis.

^af^YC-CGD: young children computer gaming disorder.

^ag^ICD-10: International Statistical Classification of Diseases, Tenth Revision.

^ah^TLI: Tucker-Lewis index.

^ai^SASC-P: Smartphone Addiction Scale-Parent version.

#### Content, Factors, and Background

Interestingly, of the 18 instruments, 7 (39%) primarily targeted behavioral patterns of children’s DT use and were named “media use” or “exposure” measures [58,60,63,79,80,84,86-88]. The other 11 (61%) instruments could be said to measure symptoms, or consequences, of a presumed underlying condition, and 7 (39%) of these were self-declared as measures of either “addiction,” “disorder,” “overdependence,” or “problematic use” [39,52,55,56,59,61,62,64,66,67,73,78,81-83,85,89,90]. Looking at the proposed factors and content categories of the instruments in Table 3, there is a great deal of variety: no 2 instruments (except Problematic Media Use Measure, which exists in a longer and shorter form) shared the same set of factors or content categories. Factors ranged from typical behavioral addiction symptoms (eg, preoccupation, tolerance, and continued use despite problems); various consequences of DT use (ie, “physical ill health,” “adverse outcomes,” and “effect on development”); behavioral patterns (ie, frequency of DT use and DT use before sleep); psychological constructs (ie, “cyberspace-oriented relationship,” “personality distortion,” and “implicit attitudes”); characteristics of the environment (ie, “digital media environment at home”); parental behaviors and attitudes (ie, parental DT use and awareness); and others. In terms of the theoretical background, 61% (11/18) of the instruments derived the item content, at least in part, from the literature on adolescents or adults.

#### Reliability and Validity

Of 18 instruments, 11 (66%) reported adequate reliability according to the values of reliability coefficients, which were >0.70, 4 (22%) did not report any measures of reliability, and 2 (11%) reported low reliability, on at least 1 factor. The most commonly used instrument (PMUM-SF) also contained the most information regarding its validity, such as structural validity, criterion validity, and measurement invariance. The information supporting the validity of all other instruments was partial at best.

## Discussion

### Shortcomings of Existing PDTU Instruments

Our scoping review showed that there are many patterns of children’s interaction with DTs beyond quantity of use, which can be seen as problematic in terms of their potential for negative consequences for children. It seems that PDTU should be understood as a multifaceted construct containing much more than excessiveness of use. A crucial observation we made during our review is the distinction between conceptualizing PDTU as a group of risky behaviors (ie, various ways of using DTs), which are likely to lead to negative outcomes, or as a group of symptoms of a presumed underlying condition (ie, addiction to DT). Existing composite measures for PDTU (ie, scales and questionnaires) have represented both conceptualizations, with the symptom view being more common. However, when proposing symptoms of a new disorder, the primary information source should, in our view, be clinical observations of multiple experienced mental health professionals working with young children. Instead, items of the reviewed PDTU instruments have mostly been generated based on reviewing the literature, commonly from established behavioral addictions in adolescents or adults, or through adapting established instruments intended for older children. In some cases, clinicians were later consulted to propose any necessary changes to items.

The PMUM-SF was the most commonly used instrument in empirical research and consists of various “symptoms” of problematic use in line with other reviews [120]. It was shown to be adequately reliable and correlated with various PDTU measures, while there is also some evidence regarding its structural validity and ability to predict relevant outcomes. However, some arguments against its validity can be made. First, its content is based on the Diagnostic and Statistical Manual of Mental Disorders–fifth edition criteria for internet gaming disorder [121], for which there is a lack of expert consensus [122]; in fact, several of them (eg, tolerance, withdrawal, and deceiving others) were later discarded from the International Classification of Diseases–11th edition definition of gaming disorder [123]. Second, the concept of PDTU extends beyond the realm of gaming and is, therefore, likely to manifest differently. Third, the criteria for internet gaming disorder were proposed for the adult or adolescent population and should not be expected to function well for the population of preschool children. Finally, Problematic Media Use Measure was originally validated on a sample of children aged 4 to 11 years, or even up to 14 years, for which significant diversity of PDTU can be expected. Unsurprisingly, some of its factors seem inappropriate for preschool children (eg, “jeopardized or lost a relationship or had compromised functioning in school due to use” and “continued use despite psychosocial problems”). Looking at the other 17 reviewed measures, both comprehensiveness and justifications for their proposed content or factor structure, as well as the presented evidence regarding the instruments’ validity, leave much to be desired. In our view, current evidence does not yet point toward a single comprehensive, reliable, and valid measurement tool for any of the 2 PDTU conceptualizations we observed in infants, toddlers, or preschool children.

### Risky Behaviors Versus Addiction Symptoms

Remaining consistent with our proposed definition of PDTU as any pattern of DT use that interferes with children’s functioning, we argue for defining PDTU through a collection of risky behaviors. This approach offers several benefits. First, focusing research efforts on developing, measuring, testing, and refining the PDTU concept in this way would yield valuable insights into the most significant ways children use DTs to their detriment. From a public health perspective, this focus has great potential to aid prevention efforts aimed at reducing the incidence of PDTU in children and the harm it causes. With high-quality evidence about the risks associated with specific DT practices, the effectiveness of awareness campaigns among parents and the screening of children for risky behaviors could be greatly improved. In contrast, adopting the addiction view of PDTU would do little to identify preventable DT practices causing harm, while screening for “addiction” symptoms would likely identify children with significant impairments in functioning, which is a notably delayed point from a prevention standpoint.

### Summary of Reviewed Single-Item Measures

#### Overview

Besides existing instruments, this review uncovered many potential single-item measures of PDTU. Notably, patterns of DT use are rarely directly observed in empirical research; instead, most (89/95, 94%) identified measures exclusively consisted of parents or caretakers recollecting their children’s behavior over a certain period. Similar findings have previously been reported [124]. Parent reports can be biased, as 1 study showed that parental reporting of coviewing, screen time limits, and active mediation were all significantly higher than the reports of their school-aged children for the same measures [125]. Still, parental reporting is considered accurate for screening and assessment of young children, especially for observable behaviors [126] and is the most practical solution for children aged 0 to 6 years. In the subsequent sections, we provide some insights regarding key PDTU measures, which should be considered by both professionals and researchers when attempting to comprehensively assess children’s PDTU.

#### Early Exposure

Measures of early exposure to DTs have been operationalized as either age of first DT use or the amount of use before a certain age (eg, screen time before the age of 1 year or 2 years). Our review examined multiple (8/95, 8%) studies where associations between early exposure and the likelihood of undesirable outcomes (eg, poor cognitive development and eyesight issues) were found, which included 2 longitudinal studies. Within the literature not included in this review, sleep problems and obesity were also highlighted as outcomes of early exposure [127]. Interestingly, higher family income and parental education were associated with earlier first exposure to DTs, contrary to the findings of Kılıç et al [128]. Considered together, these findings suggest that early exposure is one of the more evidence-based PDTU measures currently available and should be considered in any assessment of children’s PDTU.

#### Device Use Before Sleep and Devices in the Bedroom

Device use before sleep referred to the frequency of DT use before going to bed, typically within 1 hour of bedtime. Devices in the bedroom are operationalized as the number of usable digital devices present in the room where the child sleeps. Although the latter is a less-direct measure of children’s DT use, it was included in our review due to the reasoning that the presence of digital devices in the bedroom leads to a higher likelihood of unsupervised DT use or a higher likelihood of DT use before bed. In our reviewed studies, both measures were found to be associated with multiple measures of sleep quality. This finding was consistent with research showing the effect of these same PDTU measures on sleep quality in preadolescents and adolescents [129,130]. Particularly, due to the likelihood of their effect on sleep, we suggest including at least 1 of these 2 measures in the process of children’s PDTU assessment.

#### Couse or Solitary Use and Instructed Use

Cousing DTs, or rather, a lack of cousing, was one of the more common PDTU measures and refers to the extent to which DTs are used together with caregivers or siblings, sometimes as the ratio of total screen time. Cousing was positively associated with multiple measures of language development and with social development. The positive association between cousing and learning has also been found in previous studies [131]. Cousing was negatively associated with a child’s emotional liability, indicating the potential benefits of cousing DTs on the one hand and the risk of children’s solitary use on the other hand. A related PDTU measure was instructed use, that is, the frequency of parents helping children understand and navigate through digital content with the help of instructions and explanations, which can be considered a cousing measure as well. Instructed use in the first 2 years of life was associated with cognitive development at the age of 2, 3, and 4 years in a longitudinal study [32] but not with any other outcomes. On the basis of these findings, we suggest including some measure of cousing DTs into the process of assessing children’s PDTU, whether in the form of general couse or instructed use. Alternatively, the amount of unsupervised, solitary DT use may also be worth exploring as an operationalization of the same concept.

#### Device Use During Meals

Of 95 studies, 10 (11%) measured the frequency of DT use during mealtime. Only 1 (1%) study reported a positive correlation with weight, and another reported a correlation with feeding difficulties and time spent eating. The 2 other correlates (ie, difficult temperament and poor motor development) were less likely to be direct consequences of device use during meals. While these findings on preschool children were hardly conclusive, an experimental study on children aged 9 to 12 years [132] showed that children watching television while eating spent more time eating and consumed more calories than children in the control group, indicating device use during meals disrupts habituation and often leads to higher energy intake. This finding was also supported by a cross-sectional study [133]. Thus, researchers and professionals assessing PDTU may want to consider including a measure of device use during meals in the PDTU assessment process.

#### Technoference

Technoference is a relatively new concept and refers to the frequency of interruptions in parent-child interaction due to DT use. Despite being studied in only 2% (2/95) of the papers, technoference in a parent-child relationship showed significant associations with behavioral problems, psychosocial difficulties, and lower prosociality of the child. These findings are consistent with previous studies [134,135]. Importantly, 1 (1%) paper [76] distinguished between technoference due to a child’s DT use and technoference due to a parent’s DT use. In our review, only the former was considered a measure of children’s PDTU. These initial findings suggest that technoference may present an important aspect of PDTU and should be studied further.

#### Restricted Use

One of the most commonly studied single-item measures, restricted use, often called restrictive mediation, refers to parents’ use of rules and restrictions regarding their child’s DT use. Contrary to our expectations, more restricted use was associated with 2 negative rather than positive outcomes. On the other hand, restricted use was associated with less-problematic use in 2% (2/95) of the studies, one of which was longitudinal. Furthermore, some other studies suggested positive consequences of restrictive mediation [136]. We propose that these inconsistent correlations may be due to cases where parents manage to limit children’s DT use more spontaneously or due to parents being more likely to resort to explicit rules and restrictions once their children’s behavior becomes problematic. While setting explicit rules and restrictions regarding children’s DT use is generally considered good advice to parents, the absence of such explicit rules and restrictions may not work well as a measure of PDTU. Finally, this finding suggests that less-direct measures of PDTU, such as restrictive mediation, which primarily measures parent behavior, may be inadvisable.

#### Purpose of DT Use

Quite commonly, researchers asked parents and caregivers regarding the purpose for which devices were given to children (eg, for education, entertainment, regulation of a child’s emotions, or practical purposes). Using DTs for emotional regulation yielded significant longitudinal relations with a child’s emotional stability in 1 study, which was also found in studies on adolescents [137]. Because using substances or behaviors to improve one’s mood is also among the criteria for many addictions, this PDTU measure should certainly be considered and explored further. Educational use, use for entertainment, and instrumental use did not turn out to be effective predictors of negative outcomes for children, as only one such outcome was found across these 4 PDTU measures. As mentioned earlier, we propose that this is due to them being less-direct measures of children’s PDTU, through which children’s PDTU might be deduced. A potential issue with purpose-of-use measures is that despite providing insight into how children use DTs at the start of the session, the pattern of their interaction with DTs may change soon after, regardless of parents’ initial intentions.

#### Background Exposure

Only 1 (14%) out of 7 studies, which included a measure of background exposure (ie, the amount of child’s passive exposure to DTs, turned on in the background), reported associations with negative outcomes. One potential issue with this measure may be that, since children likely switch between active and passive exposure quite commonly, it is probably difficult for parents to keep track of, recollect, and accurately assess background exposure.

#### Consequences of Device Use

PDTU measures that described mostly short-term consequences of DT use, such as emotional reactivity, perceived effects of device use, conflict due to device use, and concerns about a child’s device use, did not yield any correlations with undesirable outcomes. While this is almost certainly due to the limited number of studies testing such associations, a potential issue of these variables as measures of PDTU is that the likelihood of various consequences always depends on factors beyond patterns of DT use, such as child temperament, parents’ personality traits and attitudes, and family dynamics.

#### Other Measures

For other PDTU measures, listed in Table 2, fewer or no findings regarding their associations with negative outcomes, other PDTU measures, or demographic factors were found. Since this may have been due to the lack of empirical work using these measures, more research is suggested to conclude their usefulness and predictive potential.

### Practical Implications

The findings of this review provide valuable insights into the concept of PDTU and how it should be measured and can thus serve a wide range of stakeholders, such as researchers, public mental health professionals, clinicians, educational professionals, policy makers, and others. The highlighted shortcomings of existing PDTU measures indicate the need for comprehensive, evidence-based, and standardized tools to assess early PDTU. The lack of such a tool challenges comparisons across studies and limits the development of evidence-based interventions, which has also been emphasized by other researchers [23,120]. Therefore, the results of our study can serve researchers as a basis for the development of age-appropriate measurement tools, that is, tools targeting preschool children specifically, instead of adapting measurement tools intended for adolescents or adults. Moreover, the key criteria that these tools should follow are to measure PDTU as a group of risky behaviors rather than addiction symptoms since the former provides more benefits regarding identifying and mitigating harm. Furthermore, these findings can serve as a foundation for designing informed longitudinal studies to identify causal relationships among DT use behaviors and related long-term outcomes and to better monitor PDTU in the most susceptible populations, that is, infants, toddlers, and preschool children.

Furthermore, the results of this study can serve researchers and clinicians collaborating to develop assessment tools that ensure that symptoms and behaviors reflect clinical realities. The findings can enhance screening practices and inform clinicians on how to effectively include PDTU screening in routine developmental assessments to support early identification of potentially risky behaviors (eg, bedtime use, solitary use, or the use of DT for emotional regulation). Early identification and intervention can mitigate potential negative outcomes, such as developmental delays and mental health problems. In terms of policy and public health implications, the findings of the review can inform policy makers to support and promote the establishment, implementation, and advocacy of guidelines on DT use and the development and establishment of policies promoting healthy digital habits, especially for at-risk children and families. Meanwhile, public mental health specialists can use these results to design and implement targeted public health interventions, such as awareness campaigns and programs aimed at educating families about the potential risks associated with specific DT use practices.

### Limitations

A key limitation of the findings resulting from our scoping review is the relative absence of firm evidence regarding the effects of any patterns of PDTU and certain undesirable outcomes. On the one hand, experimental and longitudinal research in this area is scarce, which means the conclusions are mainly based on correlational data from cross-sectional studies. On the other hand, even cross-sectional studies including PDTU measures beyond exposure are far from numerous for this population. Due to this, despite our recognition of the need to differentiate between findings and recommendations for infants, toddlers, and preschool children, we are currently unable to provide separate conclusions for each of these subgroups. In addition, due to our decision to only study measures of PDTU in children and due to most of these measures not including direct observation of behavior, a somewhat arbitrary decision had to be made regarding how indirectly a certain measure should target children’s behavior to still be considered a measure of PDTU. We expect that only a considerable amount of high-quality psychometric analyses will eventually reveal ways in which PDTU can be measured and how it cannot be. Furthermore, our scoping review did not include a search of gray literature, as we expected that useful findings regarding the negative outcomes of PDTU measures are much more likely to come from peer-reviewed scientific papers, although some measures may have been missed on this account. Finally, as the primary purpose of our scoping review was the identification and description of various PDTU measures, we did not conduct any risk of bias analysis. Still, some of our conclusions partly depend on the extent of evidence regarding each measure, and thus, evaluating the risk of bias in each source of evidence would have been an added benefit.

### Conclusions

In times when digital devices are increasingly accessible and used by children from infancy onward, researchers have begun to realize that screen time is likely not the optimal or sufficient way to assess the risk of troubling outcomes due to early device use. Our scoping review showed that many different measures of PDTU in early childhood exist, focusing on much more than mere exposure. While the extent of correlational evidence suggests that many patterns of DT use may be problematic, the number of displayed longitudinal effects on adverse outcomes is still insufficient to establish firm conclusions about their detrimental effects. Nevertheless, based on the findings of reviewed empirical studies, we recommend including the following measures in any assessment of children’s PDTU: early exposure to digital devices, device use before sleep or devices in the bedroom, and cousing devices or its counterpart, solitary device use. Moreover, device use during meals, device use for emotional regulation, device multitasking, and technoference may be promising PDTU measures but would benefit from more research to confirm their predictive potential. Research regarding the effects of other PDTU measures beyond exposure is still relatively modest.

This review shows that a lot of work remains to be done to establish the key problematic patterns of DT use in early childhood and how these patterns should be measured. Using reliable, valid, and comprehensive measures in this area is a prerequisite to fully assess and gain insight into the short- and long-term impact of DTs on children’s overall well-being. We urge researchers to use more quasi-experimental and longitudinal research designs, testing the effects of particular patterns of PDTU on children’s health and well-being while keeping the ethical concerns of these studies in mind.

## Appendix

Multimedia Appendix 1 A list of 95 publications examined in this scoping review and their basic characteristics.

Multimedia Appendix 2 PRISMA-ScR checklist.

## Abbreviations

DT: digital technology
PDTU: problematic digital technology use
PMUM-SF: Problematic Media Use Measure–Short Form
PRISMA-ScR: Preferred Reporting Items for Systematic Reviews and Meta-Analyses Extension for Scoping Reviews
