# Supplementary material for: Problematic Digital Technology Use Measures in Children Aged 0 to 6 Years: Scoping Review
Source: JMIR Ment Health. 2025 Mar 18;12:e59869. doi: 10.2196/59869 (PMC11962332; doi:10.2196/59869)
Supplement: Multimedia Appendix 1 [file mental_v12i1e59869_app1.docx]

Appendix A

Table A1. Characteristics of the 95 publications included in the scoping review.

| Authors, year | Country | Developmental period | Population | Sample size | Study type | Measured aspects of problematic digital technology use | Format |
| --- | --- | --- | --- | --- | --- | --- | --- |
| Durham et al., 2021 [109] | Germany | Infants | General | 630 | Cross-sectional | Early exposure, Devices in bedroom | Survey |
| Bellagamba et al., 2021 [69] | Italy | Infants, toddlers | General | 264 | Cross-sectional | Use during meals, Use before sleep, Devices in bedroom,  Emotion regulation | Survey |
| Mohapatra et al., 2020 [110] | India | Infants, toddlers | General | 105 | Cross-sectional | Devices in bedroom, Background exposure, Restricted use | Survey |
| Barber et al., 2017 [111] | UK | Infants, toddlers | General | 1558 | Longitudinal | Restricted use, Background exposure | Survey |
| Carson et al., 2012 [112] | Canada | Infants, toddlers, preschool children | General | 746 | Cross-sectional | Restricted use | Survey |
| Supanitayanon et al., 2020 [32] | Thailand | Infants, toddlers, preschool children | General | 274 | Longitudinal | Early exposure, Instructed use | Diary |
| Srisinghasongkram et al., 2020 [51] | India | Infants, toddlers, preschool children | General | 291 | Longitudinal | Device multitasking | Interview |
| Staples et al., 2021 [47] | USA | Toddlers | General | 474 | Cross-sectional | Use before sleep | Diary |
| Coyne et al., 2021 [62] | USA | Toddlers | General | 269 | Cross-sectional | Problematic use, Emotion regulation | Survey |
| Assathiany et al., 2017 [113] | France | Toddlers | General | 197 | Cross-sectional | Use during meals, Instrumental use, Emotion regulation | Survey |
| Lin et al., 2020 [114] | Taiwan | Toddlers | General | 161 | Cross-sectional | Early exposure, Educational use, Emotion regulation, Instrumental use, Co-use | Survey |
| Dy et al., 2023 [57] | Philippines | Toddlers | General | 419 | Cross-sectional | Background exposure,  Co-use | Survey |
| Teekavanich et al., 2022 [45] | Thailand | Toddlers | General | 138 | Cross-sectional | Use during meals | Survey |
| Vaidyanathan et al., 2021 [115] | India | Toddlers, preschool children | ADHD | 56 | Cross-sectional | Screen time in one sitting,  Educational use, Use for entertainment, Emotion regulation, Instrumental use | Survey |
| Emond et al., 2018 [48] | USA | Toddlers, preschool children | General | 385 | Cross-sectional | Use before sleep, Devices in bedroom | Survey |
| Almuaigel et al., 2021 [116] | Saudi Arabia | Toddlers, preschool children | General | 288 | Cross-sectional | Restricted use,  Co-use | Survey |
| Yalçın et al., 2021 [63] | Turkey | Toddlers, preschool children | General | 1245 | Cross-sectional | Delaying needs, Problematic use | Survey |
| Kristo et al., 2021 [29] | Istanbul | Toddlers, preschool children | General | 104 | Cross-sectional | Restricted use,  Use during meals | Survey |
| Yalçın et al., 2022 [54] | Turkey | Toddlers, preschool children | General | 1245 | Cross-sectional | Restricted use, Emotional reactivity,  Early exposure | Survey |
| Erat Nergiz et al., 2020 [41] | Turkey | Toddlers, preschool children | General | 138 | Cross-sectional | Early exposure, Emotion regulation, Instrumental use | Survey |
| Pons et al., 2020 [27] | Spain | Toddlers, preschool children | General | 132 | Cross-sectional | Devices in bedroom,  Use during meals, Co-use, Background exposure | Survey |
| Oflu et al., 2021 [40] | Turkey | Toddlers, preschool children | General | 240 | Cross-sectional | Early exposure, Co-use,  Emotional reactivity,  Delaying needs | Survey |
| Anitha et al., 2021 [59] | India | Toddlers, preschool children | General | 348 | Cross-sectional | Co-use, Background exposure,  Device multitasking,  Early exposure, Screen time in one sitting,  Problematic use | Survey |
| Akbayin et al., 2023 [117] | France | Toddlers, preschool children | General | 486 | Cross-sectional | Devices in bedroom,  Use before sleep, Use during meals, Co-use | Survey |
| Geng et al., 2023 [50] | China | Toddlers, preschool children | General | 126.433 | Cross-sectional | Use before sleep | Survey |
| Li et al., 2023 [65] | China | Toddlers, Preschool children | General | 1357 | Cross-sectional | Problematic use, Instrumental use | Survey |
| Rocha et al., 2023 [118] | Portugal | Toddlers, preschool children | General | 340 | Cross-sectional | Restricted use | Survey |
| Tatsiopoulou et al., 2023 [119] | Greece | Toddlers, preschool children | General | 146 | Cross-sectional | Educational use, Use for entertainment,  Co-use | Survey |
| Lin et al., 2019 [26] | Singapore | Toddlers, preschool children | Neuro-developmental disorder | 367 | Cross-sectional | Early exposure, Devices in bedroom | Survey |
| Kim et al., 2023 [37] | South Korea | Toddlers, preschool children | Social developmental delay | 96 | Cross-sectional | Early exposure, Co-use,  Emotion regulation, Background exposure | Survey |
| Fitzpatrick et al., 2022 [49] | Canada | Toddlers, preschool children | General | 316 | Cross-sectional | Use before sleep, Co-use,  Instructed use, Restricted use | Survey, diary |
| Alroqi et al., 2023 [74] | Saudi Arabia | Toddlers, preschool children | General | 85 | Cross-sectional | Early exposure, Background exposure,  Co-use,  Instructed use | Survey, diary |
| Tezol et al., 2022 [42] | Tukey | Toddlers, preschool children | General | 210 | Experimental | Early exposure | Survey |
| Ozyurt et al., 2017 [73] | Turkey | Toddlers, preschool children | Language delay and controls | 180 | Experimental | Co-use | Survey |
| Coyne et al., 2023 [56] | USA | Toddlers, preschool children | General | 432 | Longitudinal | Problematic use, Restricted use, Instructed use | Survey |
| Gueron-Sela et al., 2023 [77] | Israel | Toddlers, preschool children | General | 313 | Longitudinal | Background exposure,  Emotion regulation | Survey |
| Yildrim et al., 2023 [78] | Turkey | Toddlers, preschool children | General | 308 | Questionnaire development | Problematic use | Survey |
| Kaur et al., 2021 [79] | India | Toddlers, preschool children | General | 40 | Questionnaire development | Problematic use | Survey |
| Nikken, 2019 [71] | Netherlands | Toddlers, preschool children, school-aged children | General | NA | Cross-sectional | Instrumental use | Survey |
| Alsadoon et al., 2019 [120] | Saudi Arabia | Toddlers, preschool children, school-aged children | General | NA | Cross-sectional | Educational use, Use for entertainment, Perceived negative effects | Survey |
| Geng, Xu et al., 2023 [80] | China | Toddlers, preschool children, school-aged children | General | 711 | Cross-sectional | Early exposure, Problematic use | Survey |
| Wong et al., 2020 [75] | China | Preschool children | Disadvantaged families | 1254 | Cross-sectional | Technoference | Survey |
| Abdullah et al., 2022 [39] | Malaysia | Preschool children | General | 364 | Cross-sectional | Early exposure, Educational use, Emotion regulation, Instrumental use, Problematic use | Survey |
| Sundqvist et al., 2020 [76] | Sweden | Preschool children | General | 153 | Cross-sectional | Technoference | Survey |
| Rathnasiri et al., 2022 [121] | Sri Lanka | Preschool children | General | 340 | Cross-sectional | Early exposure, Restricted use | Survey |
| Hutton et al., 2020 [58] | USA | Preschool children | General | 47 | Cross-sectional | Problematic use | Survey |
| Wu et al., 2014 [52] | China | Preschool children | General | 202 | Cross-sectional | Use for entertainment, Educational use, Co-use,  Instructed use, Restricted use, Problematic use | Survey |
| Huang et al., 2020 [30] | China | Preschool children | General | 28,029 | Cross-sectional | Early exposure | Survey |
| Bui et al., 2022 [122] | Australia | Preschool children | General | 214 | Cross-sectional | Emotion regulation, Instrumental use | Survey |
| Chen et al., 2021 [34] | China | Preschool children | General | 29,461 | Cross-sectional | Early exposure | Survey |
| Sarı et al., 2021 [33] | Turkey | Preschool children | General | 210 | Cross-sectional | Early exposure, Background exposure,  Use for entertainment, Instrumental use | Survey |
| Yang et al., 2020 [36] | China | Preschool children | General | 26,433 | Cross-sectional | Early exposure | Survey |
| Wang et al., 2022 [46] | China | Preschool children | General | 1546 | Cross-sectional | Restricted use,  Use during meals | Survey |
| Yang et al., 2017 [53] | China | Preschool children | General | 119 | Cross-sectional | Early exposure, Co-use,  Instructed use, Restricted use | Survey |
| Munzer et al., 2018 [43] | USA | Preschool children | General | 541 | Cross-sectional | Devices in bedroom,  Use during meals, Background exposure | Survey |
| Trofholz et al., 2019 [123] | USA | Preschool children | General | 150 | Cross-sectional | Use during meals | Survey |
| Wiseman et al., 2019 [72] | Australia | Preschool children | General | 138 | Cross-sectional | Co-use,  Instructed use, Restricted use, Instrumental use | Survey |
| Park et al., 2021 [61] | South Korea | Preschool children | General | 1378 | Cross-sectional | Problematic use | Survey |
| Cho et al., 2017 [66] | South Korea | Preschool children | General | 303 | Cross-sectional | Problematic use | Survey |
| Park, 2019 [68] | South Korea | Preschool children | General | 1,378 | Cross-sectional | Problematic use | Survey |
| Felix et al., 2020 [44] | Brazil | Preschool children | General | 926 | Cross-sectional | Use during meals | Survey |
| Yang et al., 2022 [55] | Singapore | Preschool children | General | 154 | Cross-sectional | Restricted use, Problematic use | Survey |
| Li et al., 2022 [64] | China | Preschool children | General | 477 | Cross-sectional | Problematic use | Survey |
| Okano et al., 2023 [124] | Japan | Preschool children | General | 420 | Cross-sectional | Use before sleep | Survey |
| Wu et al., 2023 [125] | Taiwan | Preschool children | General | 202 | Cross-sectional | Restricted use | Survey |
| Wu et al., 2022 [38] | China | Preschool children | General | 42841 | Cross-sectional | Early exposure | Survey |
| Xiang et al., 2022 [31] | China | Preschool children | General | 4985 | Cross-sectional | Early exposure | Survey |
| Zoromba et al., 2023 [126] | Egypt | Preschool children | General | 560 | Cross-sectional | Background exposure,  Device in bedroom, Restricted use | Survey |
| Mota et al., 2019 [28] | Portugal | Preschool children | Girls | 120 | Cross-sectional | Devices in bedroom | Survey |
| Garrison et al., 2012 [127] | USA | Preschool children | General | 565 | Experimental | Co-use,  Use before sleep, Devices in bedroom | Diary, survey |
| Özyurt et al., 2018 [73] | Turkey | Preschool children | General | 76 | Experimental | Educational use, Co-use | Survey |
| Eyüboğlu et al., 2020 [35] | Turkey | Preschool children | Autism spectrum disorder and control group | 120 | Experimental | Early exposure, Perceived negative effects | Survey, interview |
| Radesky et al., 2023 [70] | USA | Preschool children | General | 422 | Longitudinal | Emotion regulation | Survey |
| Chia et al., 2019 [84] | Singapore | Preschool children | General | 137 | Questionnaire development | Problematic use | Survey |
| Hutton et al., 2020 [60] | USA | Preschool children | General | 69 | Questionnaire development | Problematic use | Survey |
| Konca et al., 2022 [85] | Turkey | Preschool children | General | 357 | Questionnaire development | Problematic use | Survey |
| Qu et al., 2021 [88] | China | Preschool children | General | 728 | Questionnaire development | Problematic use | Survey |
| Sun et al., 2022 [86] | Singapore | Preschool children | Bilingual children | 141 | Questionnaire development | Problematic use | Survey |
| Paulus et al., 2018 [90] | Germany | Preschool children, school-aged children | General | NA | Cross-sectional | Co-use, Problematic use | Survey |
| Xu et al., 2023 [87] | China | Preschool, school-aged children | General | 431 | Cross-sectional | Problematic use | Survey |
| Mukherjee et al., 2014 [128] | India | Preschool children, school-aged children | General | NA | Cross-sectional | Co-use,  Use during meals, Use before sleep | Survey |
| Mobarek et al., 2019 [129] | Egypt | Preschool children, school-aged children | General | NA | Cross-sectional | Early exposure | Survey |
| Rosen et al., 2014 [130] | USA | Preschool children, school-aged children | General | NA | Cross-sectional | Devices in bedroom | Survey |
| Saleem et al., 2014 [131] | Pakistan | Preschool children, school-aged children | General | NA | Cross-sectional | Restricted use,  Use during meals, Devices in bedroom | Survey |
| Sanders et al., 2018 [132] | USA | Preschool children, school-aged children | General | NA | Experimental | Restricted use | Survey |
| Domoff et al., 2019 [81] | USA | Preschool children, school-aged children | General | NA | Questionnaire development | Problematic use, Concerns about use | Survey |
| Domoff et al., 2021 [83] | USA | Preschool children, school-aged children, adolescents | Adverse childhood experiences | NA | Cross-sectional | Problematic use | Survey |
| Segev et al., 2015 [133] | Israel | Preschool children, school-aged children, adolescents | General | NA | Cross-sectional | Self-regulation, Restricted use | Survey |
| Harrison et al., 2021 [82] | USA | Preschool children, school-aged children, adolescents | General | NA | Cross-sectional | Problematic use, Sensory regulation, Conflict due to use | Survey |
| Ikefuna et al., 2022 [134] | Nigeria | Preschool children, school-aged children, adolescents | General | NA | Cross-sectional | Early exposure, Devices in bedroom, Restricted use | Survey |
| Eales et al., 2021 [135] | USA | Preschool children, school-aged children, adolescents | General | NA | Cross-sectional | Problematic use, Co-use,  Perceived negative effects,  Emotion regulation, Instrumental use | Survey |
| Nwankwo et al., 2019 [136] | UK | Preschool children, school-aged children, adolescents | General | NA | Cross-sectional | Concerns about use,  Restricted use | Survey, interview |
| Arora et al., 2016 [137] | USA | Preschool children, school-aged children, adolescents | Children in hospital | NA | Cross-sectional | Background exposure | Survey, observation |
| Dwairej et al., 2022 [67] | Jordan | Preschool children, school-aged children, adolescents | General | NA | Questionnaire development | Problematic use | Survey |
| Rajendhiran et al., 2021 [89] | India | Preschool children, school-aged children, adolescents | General | NA | Questionnaire development | Problematic use | Survey |

References:

26. Lin J, Magiati I, Chiong SHR, Singhal S, Riard N, Ng IH-X, Muller-Riemenschneider F, Wong CM. The Relationship Among Screen Use, Sleep, and Emotional/Behavioral Difficulties in Preschool Children with Neurodevelopmental Disorders. J Dev Behav Pediatr 2019 Sep;40(7):519–529. PMID:31107771

27. Pons M, Bennasar-Veny M, Yañez AM. Maternal Education Level and Excessive Recreational Screen Time in Children: A Mediation Analysis. Int J Environ Res Public Health 2020 Dec 1;17(23):8930. PMID:33271768

28. Mota J, Martins C, Silva-Santos S, Santos A, Vale S. TV in bedroom, outdoor playtime and obesity status among preschool girls. Science & Sports 2019 Sep;34(4):222–227. doi: 10.1016/j.scispo.2018.09.011

29. Kristo AS, Çinar N, Kucuknil SL, Sikalidis AK. Technological Devices and Their Effect on Preschool Children’s Eating Habits in Communities of Mixed Socioeconomic Status in Istanbul; a Pilot Cross-Sectional Study. Behavioral Sciences 2021 Nov 15;11(11):157. doi: 10.3390/bs11110157

30. Huang L, Yang G-Y, Schmid KL, Chen J-Y, Li C-G, He G-H, Ruan Z-L, Chen W-Q. Screen Exposure during Early Life and the Increased Risk of Astigmatism among Preschool Children: Findings from Longhua Child Cohort Study. Int J Environ Res Public Health 2020 Mar 26;17(7):2216. PMID:32224959

31. Xiang H, Lin L, Chen W, Li C, Liu X, Li J, Ren Y, Guo VY. Associations of excessive screen time and early screen exposure with health-related quality of life and behavioral problems among children attending preschools. BMC Public Health 2022 Dec 27;22(1):2440. PMID:36575397

32. Supanitayanon S, Trairatvorakul P, Chonchaiya W. Screen media exposure in the first 2 years of life and preschool cognitive development: a longitudinal study. Pediatr Res 2020 Dec;88(6):894–902. PMID:32170192

33. Sarı BA, Taner HA, Kaya ZT. Screen media exposure in pre-school children in Turkey: the relation with temperament and the role of parental attitudes. Turk J Pediatr 2021;63(5):818–831. PMID:34738364

34. Chen J-Y, Strodl E, Wu C-A, Huang L-H, Yin X-N, Wen G-M, Sun D-L, Xian D-X, Chen Y-J, Yang G-Y, Chen W-Q. Screen time and autistic-like behaviors among preschool children in China. Psychol Health Med 2021 Jun;26(5):607–620. PMID:33227216

35. Eyüboğlu M, Eyüboğlu D. Screen Time Characteristics and Early-Term Parental Concerns of Children Newly Diagnosed with Autism Spectrum Disorder. J Clin Psy 2020; doi: 10.5505/kpd.2020.23245

36. Yang G-Y, Huang L-H, Schmid KL, Li C-G, Chen J-Y, He G-H, Liu L, Ruan Z-L, Chen W-Q. Associations Between Screen Exposure in Early Life and Myopia amongst Chinese Preschoolers. Int J Environ Res Public Health 2020 Feb 7;17(3):1056. PMID:32046062

37. Kim SK, Wi DS, Kim KM. Effect of Media Exposure on Social Development in Children. Global Pediatric Health 2023 Jan;10:2333794X231159224. doi: 10.1177/2333794X231159224

38. Wu J-B, Yin X-N, Qiu S-Y, Wen G-M, Yang W-K, Zhang J-Y, Zhao Y-F, Wang X, Hong X-B, Lu D, Jing J. Association between screen time and hyperactive behaviors in children under 3 years in China. Front Psychiatry 2022 Nov 9;13:977879. doi: 10.3389/fpsyt.2022.977879

39. Abdullah NN, Mohamed S, Abu Bakar K, Satari N. The Influence of Sociodemographic Factors on Mobile Device Use among Young Children in Putrajaya, Malaysia. Children 2022 Feb 8;9(2):228. doi: 10.3390/children9020228

40. Oflu A, Tezol O, Yalcin S, Yildiz D, Caylan N, Ozdemir DF, Cicek S, Nergiz ME. Excessive screen time is associated with emotional lability in preschool children. Arch Argent Pediat 2021 Apr 1;119(2). PMID:33749196

41. Erat Nergiz M, Çaylan N, Yalçin SS, Oflu A, Tezol Ö, Foto Özdemir D, Çiçek Ş, Yıldız D. Excessive screen time is associated with maternal rejection behaviours in pre-school children. J Paediatr Child Health 2020 Jul;56(7):1077–1082. PMID:32196138

42. Tezol O, Yildiz D, Dr. Sami Ulus Training and Research Hospital Department of Pediatrics, Ankara, Turkey, Yalcin SS, Hacettepe University Faculty of Medicine Department of Social Pediatrics, Ankara, Turkey. The Psychosocial Well-Being of Young Video-Gamer Children: A Comparison Study. Turk Arch Pediatrics 2022 Jul 7;57(4):459–466. doi: 10.5152/TurkArchPediatr.2022.21359

43. Munzer TG, Miller AL, Peterson KE, Brophy-Herb HE, Horodynski MA, Contreras D, Sturza J, Lumeng JC, Radesky J. Media Exposure in Low-Income Preschool-Aged Children Is Associated with Multiple Measures of Self-Regulatory Behavior. J Dev Behav Pediatr 2018 May;39(4):303–309. PMID:29538186

44. Felix E, Silva V, Caetano M, Ribeiro MVV, Fidalgo TM, Rosa Neto F, Sanchez ZM, Surkan PJ, Martins SS, Caetano SC. Excessive Screen Media Use in Preschoolers Is Associated with Poor Motor Skills. Cyberpsychol Behav Soc Netw 2020 Jun;23(6):418–425. PMID:32511011

45. Teekavanich S, Rukprayoon H, Sutchritpongsa S, Rojmahamongkol P. Electronic media use and food intake in Thai toddlers. Appetite 2022 Sep 1;176:106121. PMID:35671917

46. Wang X, Wu Y, Yao C, Wu X, Ruan Y, Ye S. Correlates of preschoolers’ screen time in China: parental factors. BMC Pediatr 2022 Jul 14;22(1):417. PMID:35831817

47. Staples AD, Hoyniak C, McQuillan ME, Molfese V, Bates JE. Screen use before bedtime: Consequences for nighttime sleep in young children. Infant Behav Dev 2021 Feb;62:101522. PMID:33385752

48. Emond JA, Tantum LK, Gilbert-Diamond D, Kim SJ, Lansigan RK, Neelon SB. Household chaos and screen media use among preschool-aged children: a cross-sectional study. BMC Public Health 2018 Oct 29;18(1):1210. PMID:30373557

49. Fitzpatrick C, Almeida ML, Harvey E, Garon-Carrier G, Berrigan F, Asbridge M. An examination of bedtime media and excessive screen time by Canadian preschoolers during the COVID-19 pandemic. BMC Pediatr 2022 Apr 18;22(1):212. PMID:35436899

50. Geng S, Wang W, Huang L, Xie J, Williams GJ, Baker C, Du W, Hua J. Association between screen time and suspected developmental coordination disorder in preschoolers: A national population-based study in China. Front Public Health 2023;11:1152321. PMID:37050955

51. Srisinghasongkram P, Trairatvorakul P, Maes M, Chonchaiya W. Effect of early screen media multitasking on behavioural problems in school-age children. Eur Child Adolesc Psychiatry 2021 Aug;30(8):1281–1297. PMID:32856131

52. Wu CST, Fowler C, Lam WYY, Wong HT, Wong CHM, Yuen Loke A. Parenting approaches and digital technology use of preschool age children in a Chinese community. Ital J Pediatr 2014 May 7;40:44. PMID:24887105

53. Yang X, Chen Z, Wang Z, Zhu L. The Relations between Television Exposure and Executive Function in Chinese Preschoolers: The Moderated Role of Parental Mediation Behaviors. Front Psychol 2017 Oct 17;8:1833. doi: 10.3389/fpsyg.2017.01833

54. Yalçın SS, Çaylan N, Erat Nergiz M, Oflu A, Yıldız D, Tezol Ö, Çiçek Ş, Yurdakök K. Video game playing among preschoolers: prevalence and home environment in three provinces from Turkey. Int J Environ Health Res 2022 Oct;32(10):2233–2246. PMID:34260341

55. Yang H, Ng WQ, Yang Y, Yang S. Inconsistent Media Mediation and Problematic Smartphone Use in Preschoolers: Maternal Conflict Resolution Styles as Moderators. Children 2022 May 31;9(6):816. doi: 10.3390/children9060816

56. Coyne SM, Rogers A, Holmgren HG, Booth MA, Van Alfen M, Harris H, Barr R, Padilla-Walker LM, Sheppard JA, Shawcroft J, Ober M. Masters of Media: A longitudinal study of parental media efficacy, media monitoring, and child problematic media use across early childhood in the United States. J Child Media 2023;17(3):318–335. PMID:37841526

57. Dy ABC, Dy ABC, Santos SK. Measuring effects of screen time on the development of children in the Philippines: a cross-sectional study. BMC Public Health 2023 Jun 28;23(1):1261. PMID:37380949

58. Hutton JS, Huang G, Sahay RD, DeWitt T, Ittenbach RF. A novel, composite measure of screen-based media use in young children (ScreenQ) and associations with parenting practices and cognitive abilities. Pediatr Res 2020 Jun;87(7):1211–1218. PMID:32050256

59. Anitha FS, Narasimhan U, Janakiraman A, Janakarajan N, Tamilselvan P. Association of digital media exposure and addiction with child development and behavior: A cross-sectional study. Industrial Psychiatry Journal 2021 Jul;30(2):265–271. doi: 10.4103/ipj.ipj_157_20

60. Hutton JS, Dudley J, Horowitz-Kraus T, DeWitt T, Holland SK. Associations Between Screen-Based Media Use and Brain White Matter Integrity in Preschool-Aged Children. JAMA Pediatr 2020 Jan 1;174(1):e193869. PMID:31682712

61. Park JH, Park M. Smartphone use patterns and problematic smartphone use among preschool children. PLoS One 2021;16(3):e0244276. PMID:33647038

62. Coyne SM, Shawcroft J, Gale M, Gentile DA, Etherington JT, Holmgren H, Stockdale L. Tantrums, toddlers and technology: Temperament, media emotion regulation, and problematic media use in early childhood. Computers in Human Behavior 2021 Jul;120:106762. doi: 10.1016/j.chb.2021.106762

63. Yalçin SS, Tezol Ö, Çaylan N, Erat Nergiz M, Yildiz D, Çiçek Ş, Oflu A. Evaluation of problematic screen exposure in pre-schoolers using a unique tool called “seven-in-seven screen exposure questionnaire”: cross-sectional study. BMC Pediatr 2021 Oct 25;21(1):472. PMID:34696746

64. Li H, Luo W, He H. Association of Parental Screen Addiction with Young Children’s Screen Addiction: A Chain-Mediating Model. Int J Environ Res Public Health 2022 Oct 6;19(19):12788. PMID:36232109

65. Li J, Zhai Y, Xiao B, Xia X, Wang J, Zhao Y, Ye L, Li Y. Maternal COVID-19 Distress and Chinese Preschool Children’s Problematic Media Use: A Moderated Serial Mediation Model. Psychol Res Behav Manag 2023;16:2553–2567. PMID:37457390

66. Cho K-S, Lee J-M. Influence of smartphone addiction proneness of young children on problematic behaviors and emotional intelligence: Mediating self-assessment effects of parents using smartphones. Computers in Human Behavior 2017 Jan;66:303–311. doi: 10.1016/j.chb.2016.09.063

67. Dwairej DA, Obeidat HM, Alfarajat EM, Dwairej LA. Translation and Psychometric Testing of the Arabic Version of the Problematic Media Use Measure Short Form for Children. Shapka J, editor. Human Behavior and Emerging Technologies 2022 May 29;2022:1–8. doi: 10.1155/2022/4034602

68. Park JH. Factors associated with smartphone addiction risk in preschool children. 2019. doi: 10.21203/rs.2.12654/v1

69. Bellagamba F, Presaghi F, Di Marco M, D’Abundo E, Blanchfield O, Barr R. How Infant and Toddlers’ Media Use Is Related to Sleeping Habits in Everyday Life in Italy. Front Psychol 2021 Mar 22;12:589664. doi: 10.3389/fpsyg.2021.589664

70. Radesky JS, Kaciroti N, Weeks HM, Schaller A, Miller AL. Longitudinal Associations Between Use of Mobile Devices for Calming and Emotional Reactivity and Executive Functioning in Children Aged 3 to 5 Years. JAMA Pediatr 2023 Jan 1;177(1):62–70. PMID:36508199

71. Nikken P. Parents’ Instrumental use of Media in Childrearing: Relationships with Confidence in Parenting, and Health and Conduct Problems in Children. J Child Fam Stud 2019 Feb;28(2):531–546. doi: 10.1007/s10826-018-1281-3

72. Wiseman N, Harris N, Downes M. Preschool children’s preferences for sedentary activity relates to parent’s restrictive rules around active outdoor play. BMC Public Health 2019 Jul 15;19(1):946. PMID:31307424

73. Ozyurt G, Dinsever Elikucuk C. Relation of language features with maternal depression, family functioning, and digital technology usage in children with developmental language delay–comparison with healthy controls. Dusunen Adam 2017 Dec 27;299–308. doi: 10.5350/DAJPN2017300403

74. Alroqi H, Serratrice L, Cameron-Faulkner T. The association between screen media quantity, content, and context and language development. J Child Lang 2023 Sep;50(5):1155–1183. PMID:35758141

75. Wong RS, Tung KTS, Rao N, Leung C, Hui ANN, Tso WWY, Fu K-W, Jiang F, Zhao J, Ip P. Parent Technology Use, Parent-Child Interaction, Child Screen Time, and Child Psychosocial Problems among Disadvantaged Families. J Pediatr 2020 Nov;226:258–265. PMID:32629010

76. Sundqvist A, Heimann M, Koch F-S. Relationship Between Family Technoference and Behavior Problems in Children Aged 4-5 Years. Cyberpsychol Behav Soc Netw 2020 Jun;23(6):371–376. PMID:32456454

77. Gueron-Sela N, Shalev I, Gordon-Hacker A, Egotubov A, Barr R. Screen media exposure and behavioral adjustment in early childhood during and after COVID-19 home lockdown periods. Comput Human Behav 2023 Mar;140:107572. PMID:36438719

78. Yildirim M, Yayan EH. Development study of 2-5 age Technology Addiction Scale (TAS). Arch Psychiatr Nurs 2023 Apr;43:111–117. PMID:37032004

79. Kaur N, Gupta M, Kiran T, Malhi P, Grover S. Development and evaluation of the digital-screen exposure questionnaire (DSEQ) for young children. Oyeyemi AL, editor. PLoS ONE 2021 Jun 22;16(6):e0253313. doi: 10.1371/journal.pone.0253313

80. Geng S, Xu K, Liu X. Association between Electronic Media Use and Internalizing Problems: The Mediating Effect of Parent–Child Conflict and Moderating Effect of Children’s Age. Behavioral Sciences 2023 Aug 21;13(8):694. doi: 10.3390/bs13080694

81. Domoff SE, Harrison K, Gearhardt AN, Gentile DA, Lumeng JC, Miller AL. Development and validation of the Problematic Media Use Measure: A parent report measure of screen media “addiction” in children. Psychology of Popular Media Culture 2019 Jan;8(1):2–11. doi: 10.1037/ppm0000163

82. Harrison K, Couture Bue A. Media sensory curation and family media conflict: replication and validation of short-form measures. Media Psychology 2021 Jul 4;24(4):538–561. doi: 10.1080/15213269.2020.1758145

83. Domoff SE, Borgen AL, Wilke N, Hiles Howard A. Adverse Childhood Experiences and Problematic Media Use: Perceptions of Caregivers of High-Risk Youth. Int J Environ Res Public Health 2021 Jun 22;18(13):6725. PMID:34206472

84. Chia MYH, Tay LY, National Institute of Education, Nanyang Technological University, Office of Education Research, Singapore, Chua TBK, National Institute of Education, Nanyang Technological University, Physical Education and Sports Science Academic Group, Singapore. The Development of an Online Surveillance of Digital Media Use in Early Childhood Questionnaire- SMALLQ^TM^- For Singapore. Monten J Sports Sci Med 2019 Sep 1;8(2):77–80. doi: 10.26773/mjssm.190910

85. Konca AS, Baltaci Ö, Akbulut ÖF. Problematic Technology Use Scale for Young Children (PTUS-YC): Validity and Reliability Study. International Journal of Assessment Tools in Education 2022 Jun 26;9(2):267–289. doi: 10.21449/ijate.888936

86. Sun H, Lim V, Low J, Kee S. The Development of a Parental Questionnaire (QQ-MediaSEED) on Bilingual Children’s Quantity and Quality of Digital Media Use at Home. Acta Psychologica 2022 Sep;229:103668. doi: 10.1016/j.actpsy.2022.103668

87. Xu K, Geng S, Dou D, Liu X. Relations between Video Game Engagement and Social Development in Children: The Mediating Role of Executive Function and Age-Related Moderation. Behavioral Sciences 2023 Oct 11;13(10):833. doi: 10.3390/bs13100833

88. Qu F, Niu X, Huang H, Liu X. Exploratory and Confirmatory Factor Analysis of the Chinese Young Children’s Video-Gaming Questionnaire. In: Fang X, editor. HCI in Games: Experience Design and Game Mechanics Cham: Springer International Publishing; 2021. p. 266–278. doi: 10.1007/978-3-030-77277-2_21ISBN:978-3-030-77276-5

89. Rajendhiran G, Ramasubramanian V, Bijulakshmi P, Mathumathi S, Kannan M. Development and Validation of the Smartphone Addiction Scale for Children- Parent Version (SASC-P). JCDR 2021; doi: 10.7860/JCDR/2021/48398.15098

90. Paulus FW, Sinzig J, Mayer H, Weber M, Von Gontard A. Computer Gaming Disorder and ADHD in Young Children—a Population-Based Study. Int J Ment Health Addiction 2018 Oct;16(5):1193–1207. doi: 10.1007/s11469-017-9841-0

109. Durham K, Wethmar D, Brandstetter S, Seelbach-Göbel B, Apfelbacher C, Melter M, Kabesch M, Kerzel S, The KUNO Kids Study Group. Digital Media Exposure and Predictors for Screen Time in 12-Month-Old Children: A Cross-Sectional Analysis of Data From a German Birth Cohort. Front Psychiatry 2021 Nov 29;12:737178. doi: 10.3389/fpsyt.2021.737178

110. Mohapatra I, Banerjee A. Screen Dependency Disorders in children under two years of age: A Parent Report Measure. Indian J Community Health 2020 Jun 30;32(2):427–231. doi: 10.47203/IJCH.2020.v32i02.022

111. Barber SE, Kelly B, Collings PJ, Nagy L, Bywater T, Wright J. Prevalence, trajectories, and determinants of television viewing time in an ethnically diverse sample of young children from the UK. Int J Behav Nutr Phys Act 2017 Jul 6;14(1):88. PMID:28683801

112. Carson V, Janssen I. Associations between factors within the home setting and screen time among children aged 0-5 years: a cross-sectional study. BMC Public Health 2012 Jul 23;12:539. PMID:22823887

113. Assathiany R, Guery E, Caron FM, Cheymol J, Picherot G, Foucaud P, Gelbert N, Association française de pédiatrie ambulatoire, Groupe de pédiatrie générale. Children and screens: A survey by French pediatricians. Arch Pediatr 2018 Feb;25(2):84–88. PMID:29246522

114. Lin H-P, Chen K-L, Chou W, Yuan K-S, Yen S-Y, Chen Y-S, Chow JC. Prolonged touch screen device usage is associated with emotional and behavioral problems, but not language delay, in toddlers. Infant Behav Dev 2020 Feb;58:101424. PMID:32120178

115. Vaidyanathan S, Manohar H, Chandrasekaran V, Kandasamy P. Screen Time Exposure in Preschool Children with ADHD: A Cross-Sectional Exploratory Study from South India. Indian Journal of Psychological Medicine 2021 Mar;43(2):125–129. doi: 10.1177/0253717620939782

116. Almuaigel D, Alanazi A, Almuaigel M, Alshamrani F, AlSheikh M, Almuhana N, Zeeshan M, Alshurem M, Alshammari A, Mansi K. Impact of Technology Use on Behavior and Sleep Scores in Preschool Children in Saudi Arabia. Front Psychiatry 2021 May 21;12:649095. doi: 10.3389/fpsyt.2021.649095

117. Akbayin M, Mulliez A, Fortin F, Vicard Olagne M, Laporte C, Vorilhon P. Screen exposure time of children under 6 years old: a French cross-sectional survey in general practices in the Auvergne-Rhône-Alpes region. BMC Prim Care 2023 Mar 1;24(1):58. PMID:36859171

118. Rocha B, Ferreira LI, Martins C, Santos R, Nunes C. The Dark Side of Multimedia Devices: Negative Consequences for Socioemotional Development in Early Childhood. Children 2023 Nov 14;10(11):1807. doi: 10.3390/children10111807

119. Tatsiopoulou P, Holeva V, Nikopoulou VA, Parlapani E, Diakogiannis I. Changes in Sleep and Association with Screen Exposure and Diet among Preschool Children During the COVID-19 Pandemic: A Mixed Methods Study. J Child Fam Stud 2024 Feb;33(2):395–406. doi: 10.1007/s10826-023-02732-8

120. Dalal Mohammed AlSadoon NAA-N. The impact of electronic devices on children in the age group 2-12 years in Alahsa, Kingdom of Saudi Arabia. Zenodo; 2019 Jan 21; doi: 10.5281/ZENODO.2545615

121. Rathnasiri A, Rathnayaka H, Yasara N, Mettananda S. Electronic screen device usage and screen time among preschool-attending children in a suburban area of Sri Lanka. BMC Pediatr 2022 Jul 4;22(1):390. PMID:35787276

122. Bui NH, Cruickshank M, McAloon J, Maguire J. Handheld Devices: The Barrier for Parents with Mental Health Difficulties in Child Outcomes. J Child Fam Stud 2022 Jul;31(7):2027–2038. doi: 10.1007/s10826-021-02126-8

123. Trofholz AC, Tate A, Loth K, Neumark-Sztainer D, Berge JM. Watching Television while Eating: Associations with Dietary Intake and Weight Status among a Diverse Sample of Young Children. J Acad Nutr Diet 2019 Sep;119(9):1462–1469. PMID:31031108

124. Okano S, Araki A, Kimura K, Fukuda I, Miyamoto A, Tanaka H. Questionnaire survey on sleep habits of 3-year-old children in Asahikawa City: Comparison between 2005 and 2020. Brain Dev 2023 Jun;45(6):332–342. PMID:36806406

125. Wu H-Y, Lin W-Y, Huang J-P, Lin C-L, Au H-K, Lo Y-C, Chien L-C, Chao HJ, Chen Y-H. Effects of mobile device use on emotional and behavioral problems in the CBCL among preschoolers: Do shared reading and maternal depression matter? PLoS One 2023;18(7):e0280319. PMID:37450499

126. Zoromba MA, Abdelgawad D, Hashem S, El-Gazar H, Abd El Aziz MA. Association between media exposure and behavioral problems among preschool children. Front Psychol 2023;14:1080550. PMID:37546444

127. Garrison MM, Christakis DA. The impact of a healthy media use intervention on sleep in preschool children. Pediatrics 2012 Sep;130(3):492–499. PMID:22869826

128. Mukherjee SB, Gupta Y, Aneja S. Study of television viewing habits in children. Indian J Pediatr 2014 Nov;81(11):1221–1224. PMID:24682808

129. Mobarek NH, Khalil AM, Talaat DM. Exposure to Electronic Screens and Children’s Anxiety and Behavior During Dental Treatment. J Dent Child (Chic) 2019 Sep 15;86(3):139–144. PMID:31645254

130. Rosen LD, Lim AF, Felt J, Carrier LM, Cheever NA, Lara-Ruiz JM, Mendoza JS, Rokkum J. Media and technology use predicts ill-being among children, preteens and teenagers independent of the negative health impacts of exercise and eating habits. Computers in Human Behavior 2014 Jun;35:364–375. doi: 10.1016/j.chb.2014.01.036

131. Saleem M, Hassan A, Mahmood T, Mushtaq S. Factors associated with excessive TV viewing in school. Rawal Medical Journal 2014;39(3):323–326.

132. Sanders W, Parent J, Forehand R. Parenting to Reduce Child Screen Time: A Feasibility Pilot Study. J Dev Behav Pediatr 2018 Jan;39(1):46–54. PMID:28937450

133. Segev A, Mimouni-Bloch A, Ross S, Silman Z, Maoz H, Bloch Y. Evaluating Computer Screen Time and Its Possible Link to Psychopathology in the Context of Age: A Cross-Sectional Study of Parents and Children. PLoS One 2015;10(11):e0140542. PMID:26536037

134. Ikefuna AN, Uwaezuoke NA, Eze JN, Eke AL, Eke CB. Screen viewing practices and caregivers’ knowledge of the health-related effects in children and adolescents in a Nigerian Urban City. Niger J Clin Pract 2022 Jul;25(7):1115–1125. PMID:35859474

135. Eales L, Gillespie S, Alstat RA, Ferguson GM, Carlson SM. Children’s screen and problematic media use in the United States before and during the COVID-19 pandemic. Child Dev 2021 Sep;92(5):e866–e882. PMID:34486721

136. Nwankwo F, Shin HD, Al-Habaibeh A, Massoud H. Evaluation of Children’s Screen Viewing Time and Parental Role in Household Context. Glob Pediatr Health 2019;6:2333794X19878062. PMID:31579685

137. Arora G, Soares N, Li N, Zimmerman FJ. Screen Media Use in Hospitalized Children. Hosp Pediatr 2016 May;6(5):297–304. PMID:27076442
